# Chromosome level genome assembly of the World Health standards *Leishmania (Viannia) **guyanensis*** M4147 and *L. (V.) **shawi*** M8408 using a hybrid sequencing approach

**DOI:** 10.1590/0074-02760250270

**Published:** 2026-06-19

**Authors:** Percy Omar Túllume-Vergara, Ana Carolina Stocco de Lima, Claudia Maria de Castro Gomes, Bruna Santos Lima, Sinval Pinto Brandão-Filho, Fernando Tobias Silveira, Jeffrey J Shaw, João Marcelo Pereira Alves

**Affiliations:** 1 Universidade de São Paulo, Instituto de Ciências Biomédicas, Departamento de Parasitologia, São Paulo, SP, Brasil; 2 Instituto Evandro Chagas, Departamento de Parasitologia, Ananindeua, PA, Brasil; 3 Universidade de São Paulo, Faculdade de Medicina, Departamento de Patologia, São Paulo, SP, Brasil; 4 Fundação Oswaldo Cruz-Fiocruz, Instituto Aggeu Magalhães, Recife, PE, Brasil; 5 Universidade Federal do Pará, Núcleo de Medicina Tropical, Belém, PA, Brasil

**Keywords:** genome assembly, Leishmania **guyanensis**, Leishmania **shawi**, subgenus *Viannia*, phylogenomics

## Abstract

**BACKGROUND:**

The *Leishmania* (*Viannia*) subgenus contains important pathogens that cause a variety of different clinical forms of cutaneous leishmaniasis in the Americas. Their response to antimonial chemotherapy differs according to species. Having high-quality genomic resources of these species is a significant step towards investigating and understanding these factors.

**OBJECTIVES:**

This study aims to characterise the main genomic features of *L. (V.) **guyanensis*** strain MHOM/BR/75/M4147 and *L. (V.) **shawi*** strain MCEB/BR/84/M8408.

**METHODS:**

Genomes were sequenced combining short- and long-read sequencing platforms and assembled, scaffolded, and polished using Flye2, Ragtag, and Pilon, respectively. Annotations were performed using mainly similarity and profile search methods, and phylogenetic analyses were performed using the maximum likelihood (ML) and Bayesian inference approaches, using IQ-TREE and MrBayes, respectively.

**FINDINGS:**

*De novo* assembly produced genome sizes of 32.27 Mb for *L. **guyanensis*** and 32.41 Mb for *L. **shawi***, and predicted 8,505 and 8,592 protein-coding genes, respectively. Phylogenetic analysis based on these assemblies confidently places *L. **guyanensis*** and *L. **shawi*** as the closest known relatives to *L. panamensis* within the *Viannia* clade.

**MAIN CONCLUSIONS:**

These genomes will increase the knowledge about the subgenus *L.* (*Viannia*) in the Americas and also represent valuable information for future comparative studies with other human pathogenic *Leishmania* spp.

Leishmaniinae parasites of the subgenus *Leishmania* (*Viannia*) cause a wide range of cutaneous and mucosal leishmaniasis and are only found in Central and South America. The subgenus was created in 1987^1^ and presently comprises nine species.^2^ There is some evidence that the severity of the pathology due to *L.* (*V.*) *braziliensis* and *L.* (*V.*) *guyanensis* in some geographical regions is associated with the presence of high *Leishmania RNA virus 1* (LRV1) burden.^3^

*Leishmania* (*V*.) *guyanensis* (MHOM/BR/75/M4147) and *L.* (*V.*) *shawi* (MCEB/BR/84/M8408) are World Health Organization (WHO) standards and M8408 cultures are hapanotypes of the species. M8408 was isolated from a brown capuchin monkey (now classified as *Sapajus apella*) that was captured in 1984 in the Carajá Mountains of Pará state, Brazil. Both species are important pathogens and are the dominant species identified in man in some Amazonian regions.^4^

*Leishmania* (*V*.) *guyanensis* occurs in a vast region to the north of the Amazon River. In 2000, 100% of the cases from Manaus^5,6^ were caused by *L.* (*V*.) *guyanensis*, but 10 years later they represented 73% of the cases. In the Guiana Shield and the Guianas it is involved in 80-94% of the cases.^6^ *L.* (*V*.) *shawi* occurs in regions south of the Amazon River. In the Santarém microregion, it accounts for 26% of the cases,^7^ while another 17% were caused by *guyanensis*/*shawi* hybrids. *L.* (*V*.) *shawi* was not recorded in the Belém Metropolitan region,^8^ but in the past cases have been recorded in the southeastern regions of Pará State.^9^ Over time, the variations seen in species prevalence are most probably due to environmental changes that influence vector and reservoir host distribution.

Previous versions of draft assemblies generated using short reads for *L.* (*V*.) *guyanensis* have been published for strains LgCL085,^10^ MHOM/GF/2004/204-365,^11^ and MHOM/BR/75/M4147.^12^ In this study, we have sequenced, assembled and analysed chromosome-level genome assemblies of *L.* (*V.*) *guyanensis* and *L.* (*V.*) *shawi*. We analysed these *Viannia* species to identify key genomic features and establish their phylogenetic relationships.

## MATERIALS AND METHODS

Whole-genome sequencing was performed using two technologies, Illumina (short reads) and Pacific Biosciences-PacBio (long reads), and genomes were assembled using a hybrid approach. Genomic DNA was extracted by the phenol-chloroform method from promastigote cultures in the log phase using standard methodologies.^13^ We also assessed the quality of the reads using Fastp v.0.20.1.^14^

Both genomes were assembled using a *de novo* approach, using only high-quality long reads and the Flye2 v.2.9 assembler,^15^ with option "genomeSize=35Mb". To elevate the draft Flye2 assemblies to pseudochromosome level, we performed scaffolding with Ragtag v.2.1.0.^16^ The *L. (V.) **braziliensis*** strain M2904 genome^17^ served as the reference, chosen because it is a close relative of our *Viannia* species^18^ and is itself correctly assembled into the 35 chromosomes, being one the most complete references to date. To polish (*i.e.*, correct inconsistencies) in the final assemblies, we ran Pilon v.1.24^19^ using quality-filtered reads from the Illumina library, with the "--fix_all" option. The polishing cycle was repeated three times to minimise errors and produce a high-accuracy consensus sequence. kDNA was identified using BLASTN against a custom mitochondrial database (generated by collecting previously sequenced trypanosomatid mtDNA from several species). Telomeric repeats in the *L. (V.) **guyanensis*** and *L. (V.) **shawi*** genomes were systematically identified using the tidk v. 0.2.7 with the search module^20^ and using the canonical trypanosomatid motif ("TTAGGG"). Some telomeric regions were detected on only a subset of chromosomal ends [Supplementary data (Table I)], indicating incomplete reconstruction for these repeats in the assembly process. Assessment of assembly completeness was performed with BUSCO v. 5.3.1,^21^ using the lineage Euglenozoa DataBase release 10 (130 single-copy orthologs) and the "-m genome" option.

Furthermore, to conduct a comprehensive genome-wide comparison, we used the D-GENIES webtool v. 1.5.0^22^ with both whole-genome assemblies. Gene structure prediction was performed using AUGUSTUS v.3.3.3, using the "intronless" option, except for three genes with introns that were manually annotated.^23^ The resulting protein sequences were annotated using InterProScan v. 5.63-95.0.^24^ Non-coding RNAs sequences were predicted and annotated using INFERNAL v.1.1.4^25^ with Rfam v.14.9^26^ database as a reference, along with tRNAscan-SE v.2.0.3.^27^

Genomic synteny was assessed using SyRI v.1.7.1.^28^ Single-copy orthologous genes (n = 3,962) were identified from all proteomes using OrthoFinder v. 2.5.4.^29^ The corresponding protein sequences were aligned using MUSCLE v. 3.8.31,^30^ and ambiguously aligned regions (gaps) were removed using TrimAl v. 1.4 with the "automated_1" option.^31^ The resulting alignments were concatenated into a supermatrix using FASconCAT-G v. 1.0.^32^ Phylogenies were reconstructed with both maximum likelihood (ML) and Bayesian inference (IB) approaches, employing IQ-TREE v. 2.0.4^33^ and MrBayes v. 3.2.6,^34^ respectively. For the ML analysis, the optimal substitution model for each partition was selected by ModelFinder.^35^ Node support was assessed using bootstrap values (ML) and posterior probabilities (BI), with BI supports ≥ 0.95 considered robust. Trees were visualised and annotated in iTOL v. 5.4 (Available from: https://itol.embl.de/),^36^ accessed on 30/03/2025.

## RESULTS

**Fig. 1:**
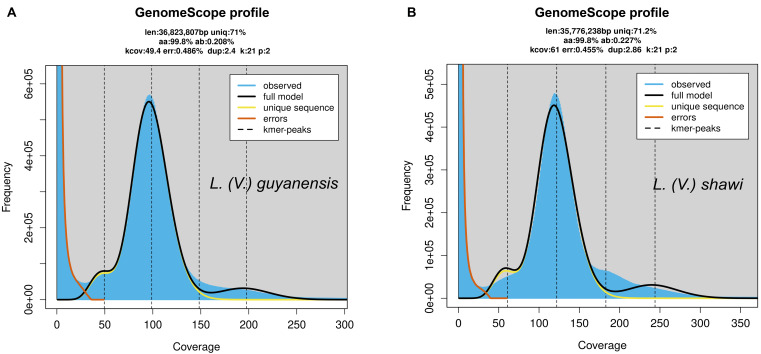
comparison of genome profile between *Leishmania (Viannia) **guyanensis*** (A) and *L. (V.) **shawi*** (B). Results of genome size (GS) estimation of both genomes obtained by GenomeScope k-mer distribution using kmer length = 21 for short reads. len: inferred genome length (GS); uniq: % of the genome that is unique; het: overall rate of heterozygosity; dup: average rate of read duplications.

In the *L.* (*V*.) *guyanensis* M4147 strain, the Illumina NextSeq library consisted of 19,159,530 paired-end reads with a length of 131 bp, and the single-molecule real-time (SMRT) library PacBio platform produced 638,748 reads with an average size of 9,087 bp. In *L.* (*V*.) *shawi*, 20,703,763 paired-end reads of 131 bp in length were sequenced with Illumina NextSeq, and the SMRT library was generated with 160,534 reads of an average of 7,565 bp in length (Table I). In order to estimate features such as genome size (GS), heterozygosity, and repetitiveness independently of assembled sequences, Jellyfish v. 2.2.10 and GenomeScope 2.0 were used,^37^,^38^ with results shown in Fig. 1A and 1B.

**TABLE I:** The comparison of genomic read statistics between *Leishmania (Viannia) **guyanensis*** M4147 and *L. (V.) **shawi*** M8408 strain

Features	*L. (V.) **guyanensis***	*L. (V.) **shawi***
Illumina paired-end raw reads	19,159,530	20,703,763
Pacbio raw reads	663,350	167,720
Size for Illumina paired-end (bp)	131	131
Average size for Pacbio (bp)	9,087	7,565
Estimate of genome size (bp)	33,368,794	35,776,238
Genome coverage (Pacbio)	167x	36x
Genome coverage (Illumina)	133.44x	172.47x

A dot plot showing macrosynteny between *L. (V.) **guyanensis*** and *L. (V.) **shawi*** is shown in [Supplementary data (Fig. 1)]. A detailed overview of the generated sequences, assembly statistics, and completeness values is shown in Table II. Table II shows a total of 8,505 and 8,592 protein-coding genes were annotated for *L. (V.) **guyanensis*** and *L. (V.) **shawi***, respectively. Prediction of non-coding RNA (NCrna) genes revealed several types of RNAs that ranged from 321 to 354 sequences; detailed counts for each type are provide in Table II.

**TABLE II:** Assembly quality and gene prediction comparison among species of the *Leishmania* (*Viannia*) subgenus

Features	*L.* (*V*.) *guyanensis* M4147	*L.* (*V*.) *shawi* M8408	*L.* (*V*.) *braziliensis* M2904 ^17^	*L.* (*V*.) *panamensis* PSC-1 ^41^
Number of pseudochromosomes	35	35	35	35
Total genome assembly size (Mb)	32.27	32.41	32.30	30.69
GC content (%)	57.83	57.69	57.72	57.56
Number of contigs	47	64	35	35
Scaffold N50	1 Mb	929.9 Kb	1Mb	1 Mb
Assembly size of mtDNA (maxicircle)	25.5Kb	15.8 Kb	-	-
BUSCO [Euglenozoa ODB.10]	130 [Complete]	130 [Complete]	130 [Complete]	130 [Complete]
Protein-coding genes	8,505	8,592	8,588	8,246
Transfer RNA	78	98	78	74
Ribosomal RNA	15	15	8	17
Small nucleolar RNA	261	208	32	54

(-): no information.

**Fig. 2:**
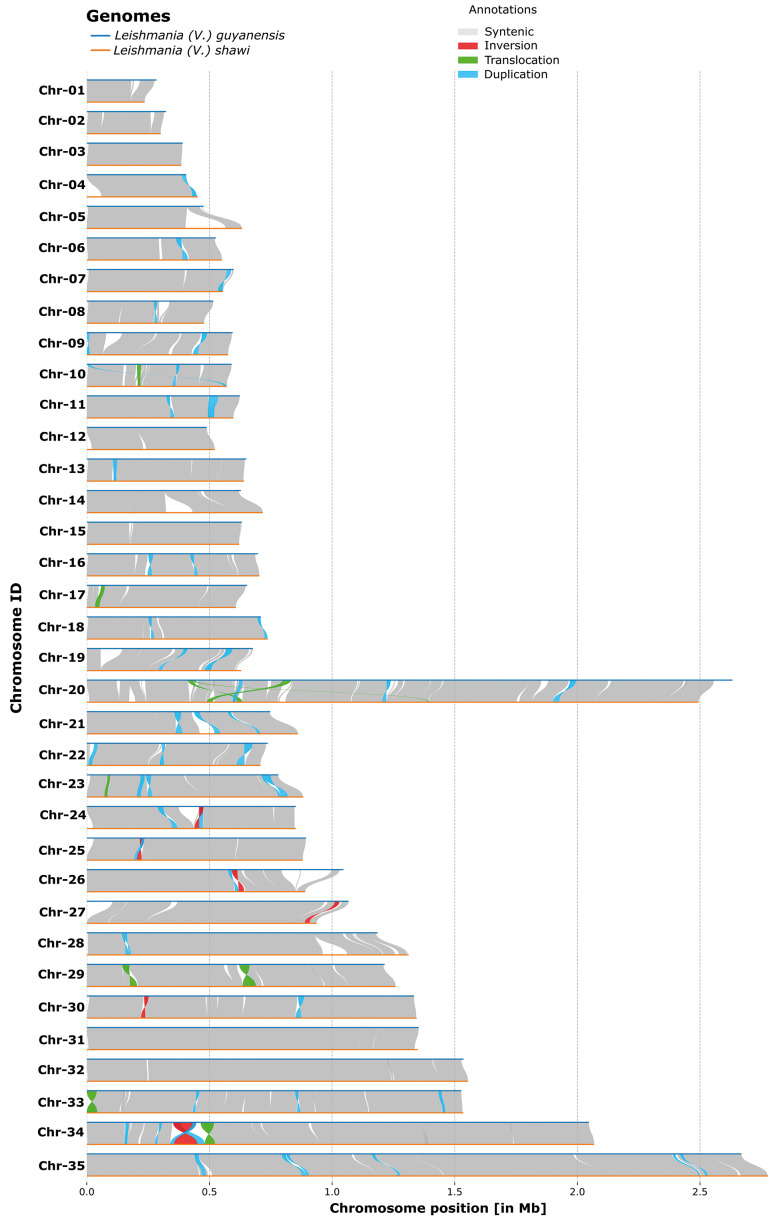
structural variants assessed by SyRI between *Leishmania (Viannia) **guyanensis*** (top-reference) and *L. (V.) **shawi*** (bottom-query) genome assemblies. This figure displays the 35 chromosomes pair by pair, with synteny regions coloured in grey lines and structural rearrangements coloured as follows: duplications (sky blue), inversions (red), and translocations (green).

The level of synteny between the genomes of *L. (V.) **guyanensis*** (reference) and *L. (V.) **shawi*** (target) was assessed at the nucleotide level. The analysis revealed a strong collinearity and a minimum of rearrangement between the two species, with 231 syntenic regions detected (Fig. 2). Among these, 37 translocations and 11 inversions were identified. Additionally, 301 and 133 duplications were counted in *L. (V.) **guyanensis*** and *L. (V.) **shawi***, respectively. SyRI-based variant calling detected 150,032 single-nucleotide differences and 54 tandem repeat sequences between the two genomes. While most chromosomes exhibited strong synteny (Fig. 2), structural variations were observed including inversions in chromosomes 24, 25, 26, 27, 30, and 34. Another chromosomal structural mutation observed was translocation, affecting chromosomes 10, 17, 20, 23, 29, 33, and 34. Despite the overall conservation across all 35 chromosomes, SyRI also revealed multiple structural rearrangements when comparing the genomes of *L. **guyanensis*** and *L. **shawi*** (Fig. 2). Additionally, chromosome copy number variation (CCNV) was estimated by read depth coverage (RDC) following the protocol detailed in Briggs et al.,^39^ which assumed that the genomes are diploid (2n). CCNV revealed that most chromosomes in *L. (V.) **guyanensis*** and *L. (V.) **shawi*** are predominantly diploid, except for chromosome 31, which exhibits copy numbers between trisomy and tetrasomy [Supplementary data (Fig. 2)].

**Fig. 3:**
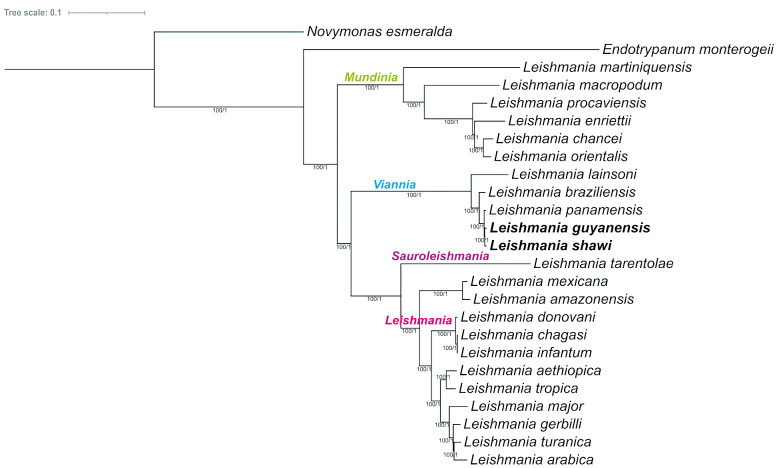
maximum-likelihood (ML) and Bayesian inference (BI) phylogenomic trees based on the supermatrix of 3,962 proteins encoded by single-copy genes. BI and ML methods yield identical topologies; both trees are shown. The values above branches are bootstrap values together with posterior probabilities (see Materials and Methods). The bar indicates number of substitutions per site.

To determine the phylogenetic position of *L. (V.) **guyanensis*** and *L. (V.) **shawi*** within the genus, we performed a phylogenomic analysis of 25 reference genomes, incorporating two outgroup taxa (*Novymonas esmeralda* and *Endotrypanum monterogeii* [Supplementary data (Table II)]. The resulting phylogeny (Fig. 3) robustly placed *L. (V.) **guyanensis*** and *L. (V.) **shawi*** within the *Viannia* subgenus, as expected. Their closest relative was *L. (V.) panamensis*. The outgroup species were positioned on early-diverging branches, with *E. monterogeii* exhibiting a long branch length indicative of its previously reported elevated evolutionary rate.^40^ The tree topology was strongly supported, with most nodes receiving 100% ML bootstrap support (Fig. 3).

## DISCUSSION

Leishmaniasis poses a significant threat to human health, yet the genomic resources of its causative parasites are inadequately explored in Latin America. The isolates M4147 of *L. (V.) **guyanensis*** and M8408 of *L. (V.) **shawi*** are of paramount importance as WHO reference standards, since high-quality genomes are lacking for the two species. Existing assemblies are frequently highly fragmented, a limitation that propagates errors in fundamental genomic features including genome size, copy number analysis, repetitive element characterisation, and gene annotation.^10,11,12^ Therefore, generating complete and high-fidelity genomes for these strains is crucial to empower accurate comparative and functional studies across the *Leishmania* research field. In this study, we provide genome assemblies of *L. (V.) **guyanensis*** and *L. (V.) **shawi*** using a hybrid approach that produced significant improvements in contiguity and completeness, as indicated by high N50 values and complete BUSCO scores. The high quality of our assemblies is validated by their parity with the reference genomes of key *Viannia* species, *L. (V.) **braziliensis*** and *L. (V.) **panamensis***.^17,41^

Our analysis of chromosomal somy revealed that while both species are predominantly diploid, they also exhibit consistent aneuploidy. Specifically, chromosome 31 appears to be present in supernumerary copy numbers. This finding aligns with prior reports of tetrasomy on chromosome 31 in other *Leishmania* species, including *L. **guyanensis*** ^42^ and *L. panamensis* ^43^ within the *Viannia* subgenus, as well as in *L. **donovani*** and *L. major* of the *Leishmania* subgenus.^44^,^45^ Aneuploidy is a well-documented driver of adaptive evolution in *Leishmania*, where changes in chromosome copy number are frequently associated with drug resistance,^43^,^46^ environmental adaptation,^47^ and virulence.^48^ Given these established links and the recurrent observation of chromosome 31 across diverse species, we hypothesise that this tetrasomy confers a significant selective advantage, likely by modulating gene dosage to certain cellular processes, such as an environment with oxidative stress.^49^

Based on an analysis of 25 whole genomes, our phylogenomic reconstruction indicates that *L. (V.) **guyanensis*** and *L. (V.) **shawi*** are the most recently diverged species within the *Viannia* clade. Both ML and BI methods consistently supported this phylogenetic placement, yielding congruent topologies. Although our tree is consistent with prior studies,^20,49^ the employment of 3,962 single-copy genes revealed only short branch length between these species, indicating very limited phylogenetic divergence. This similar pattern was also observed between *L. (L.) infantum* and *L. (L.) **chagasi***.^50^

Due to this shallow genetic separation and ongoing taxonomic uncertainty regarding *L. (V.) **shawi***, which has been proposed to belong to the *L. (V.) **guyanensis*** species complex alongside *L. **panamensis***,^18^,^51^ we recommend that future investigations incorporate broader species sampling and complementary approaches such as phylogenetic networks or genome-wide single nucleotide polymorphisms (SNP) analysis. Hence, these methods could better resolve potential lack of evolutionary signals and clarify whether *L. (V.) **guyanensis*** and *L. (V.) **shawi*** represent distinct species or a single genetic lineage. Our present analysis cannot definitively resolve this question.

Although not fully telomere-to-telomere complete [Supplementary data (Table I)], the genome assemblies presented here are the most comprehensive currently available for these *Leishmania* species, offering a critical genomic resource for the study of their biology.

In summary, we present two newly sequenced chromosome-level genomes of *L. (V.) **guyanensis*** and *L. (V.) **shawi***, along with their main analysis including assembly, annotations, synteny assessment, and phylogenomics analysis. We also identified a putative tetrasomy of chromosome 31 in both isolates. Furthermore, phylogenomic analysis confirms that these recently diverged species form a monophyletic clade with *L. (V.) panamensis*. These two high-quality genomes generated here enhance our understanding of *Leishmania* genome biology, providing valuable resources for future research of the *Viannia* subgenus.

## SUPPLEMENTARY MATERIALS

Supplementary data

## Data Availability

The raw sequence reads of Illumina and PacBio platforms are available under BioProject accession number PRJNA1167797. The *Leishmania **guyanensis*** and *L. **shawi*** genome assemblies are available under accession numbers JBIEOV000000000 and JBINZM000000000, respectively.
