# Supplementary material for: Chromosome level genome assembly of the World Health standards Leishmania (Viannia) guyanensis M4147 and L. (V.) shawi M8408 using a hybrid sequencing approach
Source: Mem Inst Oswaldo Cruz. 2026 Jun 19;121:e250270. doi: 10.1590/0074-02760250270 (PMC13281984; doi:10.1590/0074-02760250270)
Supplement: Supplementary data [file 1678-8060-mioc-121-e250270-s1.pdf]

TABLE I

Telomere repeat occurrence for *Leishmania (Viannia) guyanensis* and *L. (V.) shawi*

Chromosome	Telomere repeat number			
	L. guyanensis 5'	*L. guyanensis* 3'	*L. shawi* 5'	*L. shawi* 3'
Chr01	31	1	2	2
Chr02	574	0	9	3
Chr03	6	1	6	0
Chr04	4	2	162	3
Chr05	5	0	19	0
Chr06	2	2	292	2
Chr07	22	8	8	12
Chr08	0	0	1	1
Chr09	4	3	3	0
Chr10	0	0	0	0
Chr11	2	2	1	0
Chr12	2	3	9	1
Chr13	28	2	6	0
Chr14	2	2	6	1
Chr15	2	7	0	0
Chr16	6	9	0	0
Chr17	2	7	1	0
Chr18	8	2	0	0
Chr19	1	1	5	306
Chr20	0	0	2	2
Chr21	0	5	5	0
Chr22	5	6	2	0
Chr23	23	2	3	2
Chr24	2	5	0	1
Chr25	0	0	0	0
Chr26	170	1	2	1
Chr27	3	1	0	0
Chr28	3	32	0	0
Chr29	4	1	5	7
Chr30	6	11	4	32
Chr31	0	0	3	15
Chr32	2	8	0	0
Chr33	1	2	3	0
Chr34	1	19	2	26
Chr35	0	0	1	0

TABLE II

List of 23 reference genomes employed for phylogenomic reconstruction. New assembly genomes are displayed in bold

Species name	Strain	Life cycle	# of proteins	References
Leishmania chancei	LV757	Dixenous	8,119	(1)
Leishmania enriettii	LEM3045	Dixenous	8,731	(2)
Leishmania orientalis	LSCM4	Dixenous	8,158	(2)
Leishmania procaviensis	LV425	Dixenous	8,266	(3)
Leishmania mexicana	U1103	Dixenous	8,144	(4)
Leishmania macropodum	AM-2004	Dixenous	7,935	(5)
Leishmania major	Friedlin	Dixenous	8,424	(6)
Leishmania infantum	JPCM5	Dixenous	8,527	(7)
Leishmania tarentolae	Parrot Tar II	Dixenous	8,703	(8)
Leishmania lainsoni	216-34	Dixenous	8,959	(9)
Leishmania donovani	BPK282A1	Dixenous	7,969	(10)
Leishmania amazonensis	M2269	Dixenous	8,481	(11)
Leishmania arabica	LEM1108	Dixenous	8,646	(12)
Leishmania gerbilli	LEM452	Dixenous	8,599	(12)
Leishmania martiniquensis	LEM2494	Dixenous	8,483	(12)
Leishmania tropica	L590	Dixenous	8,824	(12)
Leishmania turanica	LEM423	Dixenous	8,608	(12)
Novymonas esmeralda	E262AT	Monoxenous	9,837	(13)
Leishmania braziliensis	M2904	Dixenous	8,484	(19)
Leishmania panamensis	PSC-1	Dixenous	7,748	(41)
Leishmania chagasi	M6445	Dixenous	8,409	(50)
Leishmania guyanensis	**M4147**	**Dixenous**	**8,505**	**This study**
Leishmania shawi	**M8408**	**Dixenous**	**8,592**	**This study**

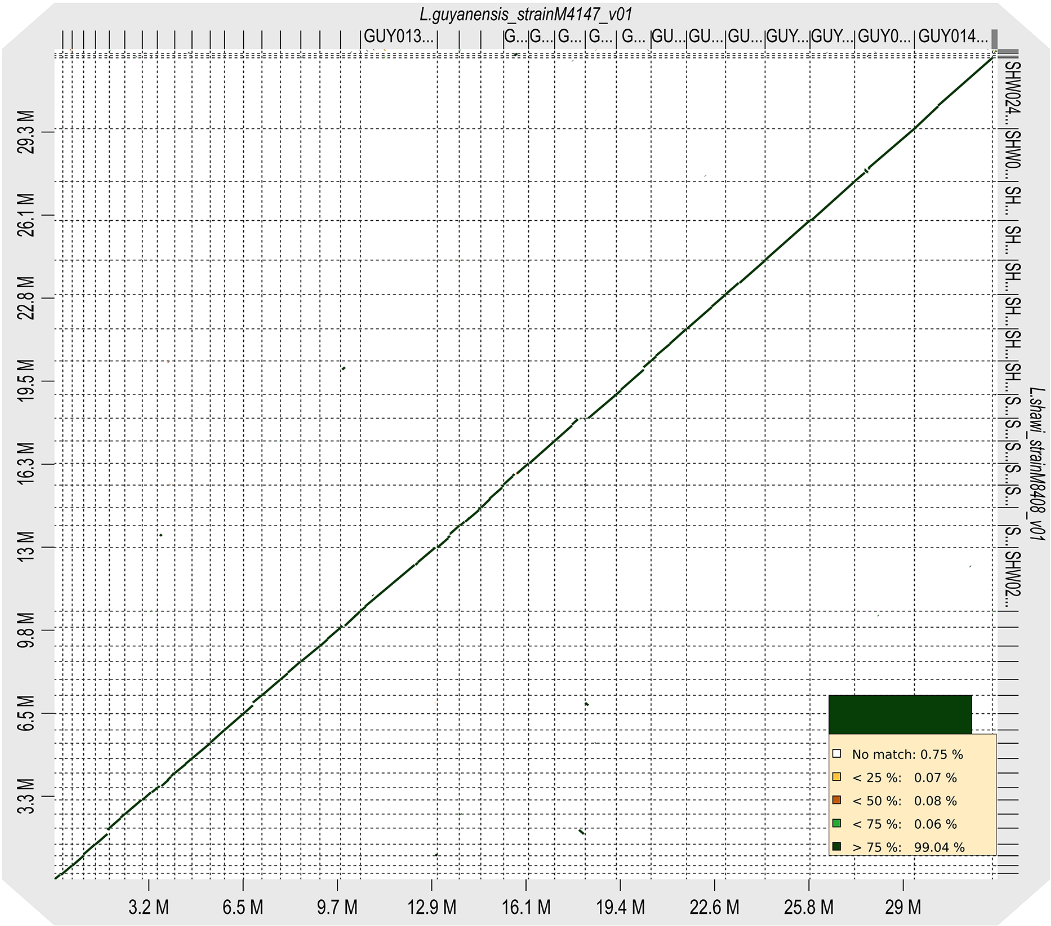

Fig. 1: dot-plot built using the D-Genies platform to compare the similarity of *Leishmania (Viannia) guyanensis* (horizontal axis) versus *L. (V.) shawi* (vertical axis) genome assemblies. Dotted gridlines account for chromosome boundaries. The colours in the dot-plot represent identity levels.

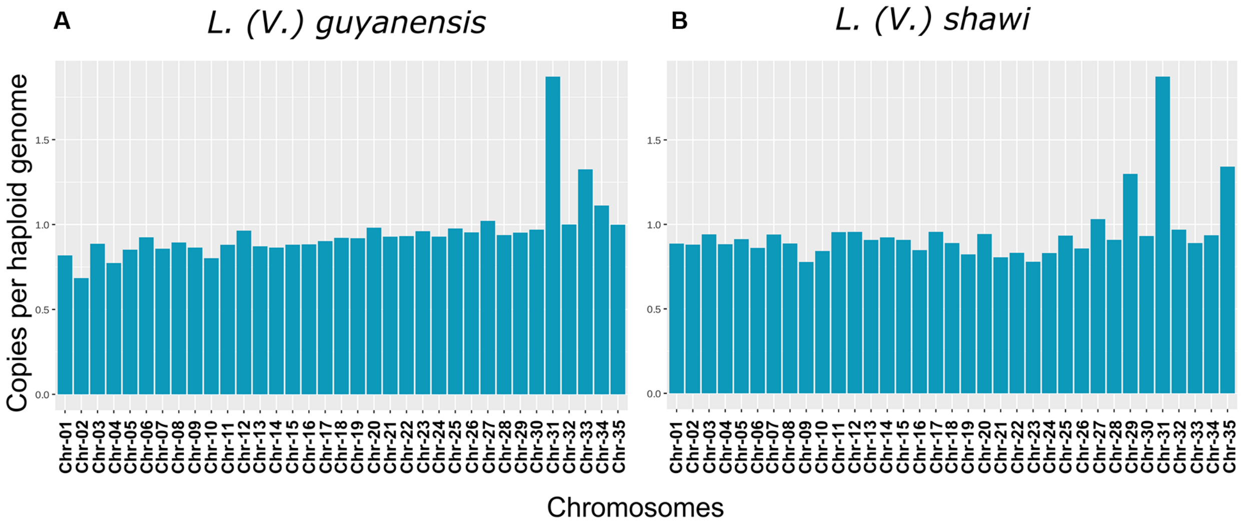

Fig. 2: chromosome copy number estimation by read depth coverage (RDC). Chromosome somy estimated based on RDC in *Leishmania (Viannia) guyanensis* and *L. (V.) shawi*. The x-axis represents all 35 chromosomes of *Leishmania* spp. The y-axis shows the median gene coverage per chromosome, normalised by genome-wide coverage (RDC). Median RDC values of 0.5, 1.0, and 2 denote that the chromosome has, respectively, “0.5”, “1”, or “2” copies per haploid genome.

REFERENCES

Almutairi H, Urbaniak MD, Bates MD, Kwakye-Nuako G, Al-Salem WS, Dillon RJ, et al. Chromosome-scale assembly of the complete genome sequence of *Leishmania (Mundinia)* sp. Ghana, isolate GH5, strain LV757. Microbiol Resour Announc. 2021; 10(39): e0059121. doi: 10.1128/MRA.00591-21.

Almutairi H, Urbaniak MD, Bates MD, Jariyapan N, Kwakye-Nuako G, Thomaz Soccol V, et al. Chromosome-scale genome sequencing, assembly and annotation of six genomes from subfamily Leishmaniinae. Sci Data. 2021; 8(1): 234. doi: 10.1038/s41597-021-01017-3.

Almutairi H, Urbaniak MD, Bates MD, Kwakye-Nuako G, Al-Salem WS, Dillon RJ, et al. Chromosome-scale assembly of the complete genome sequence of *Leishmania (Mundinia) procaviensis* isolate 253, strain LV425. Microbiol Resour Announc. 2023; 12(4): e0130622. doi: 10.1128/mra.01306-22.

Batra D, Lin W, Narayanan V, Rowe LA, Sheth M, Zheng Y, et al. Draft genome sequences of *Leishmania (Leishmania) amazonensis*, *Leishmania (Leishmania) mexicana*, and *Leishmania (Leishmania) aethiopica*, potential etiological agents of diffuse cutaneous leishmaniasis. Microbiol Resour Announc. 2019; 8(20): e00269-19. doi: 10.1128/MRA.00269-19.

Butenko A, Kostygov AY, Sádlová J, Kleschenko Y, Bečvář T, Podešvová L, et al. Comparative genomics of *Leishmania (Mundinia)*. BMC Genomics. 2019; 20(1): 726. doi: 10.1186/s12864-019-6126-y.

Camacho E, González-de la Fuente S, Solana JC, Rastrojo A, Carrasco-Ramiro F, Requena JM, et al. Gene annotation and transcriptome delineation on a *De Novo* genome assembly for the reference *Leishmania major* Friedlin strain. Genes (Basel). 2021; 12(9): 1359. doi: 10.3390/genes12091359.

González-de la Fuente S, Peiró-Pastor R, Rastrojo A, Moreno J, Carrasco-Ramiro F, Requena JM, et al. Resequencing of the *Leishmania infantum* (strain JPCM5) genome and *de novo* assembly into 36 contigs. Sci Rep. 2017; 7(1): 18050. doi: 10.1038/s41598-017-18374-y.

Goto Y, Kuroki A, Suzuki K, Yamagishi J. Draft genome sequence of *Leishmania tarentolae* Parrot Tar II, obtained by single-molecule real-time sequencing. Microbiol Resour Announc. 2020; 9(21): e00050-20. doi: 10.1128/MRA.00050-20.

Lin W, Batra D, Narayanan V, Rowe LA, Sheth M, Zheng Y, et al. First draft genome sequence of *Leishmania (Viannia) lainsoni* strain 216-34, isolated from a Peruvian clinical case. Microbiol Resour Announc. 2019; 8(6): e01524-18. doi: 10.1128/MRA.01524-18.

Lypaczewski P, Hoshizaki J, Zhang WW, McCall LI, Torcivia-Rodriguez J, Simonyan V, et al. A complete *Leishmania donovani* reference genome identifies novel genetic variations associated with virulence. Sci Rep. 2018; 8(1): 16549. doi: 10.1038/s41598-018-34812-x.

Real F, Vidal RO, Carazzolle MF, Mondego JM, Costa GG, Herai RH, et al. The genome sequence of *Leishmania (Leishmania) amazonensis*: functional annotation and extended analysis of gene models. DNA Res. 2013; 20(6): 567-81. doi: 10.1093/dnares/dst031.

Warren WC, Akopyants NS, Dobson DE, Hertz-Fowler C, Lye LF, Myler PJ, et al. Genome assemblies across the diverse evolutionary spectrum of *Leishmania* protozoan parasites. Microbiol Resour Announc. 2021; 10(35): e0054521. doi: 10.1128/MRA.00545-21.

Zakharova A, Saura A, Butenko A, Podešvová L, Warmusová S, Kostygov AY, et al. A new model trypanosomatid, *Novymonas esmeraldas*: genomic perception of its "Candidatus Pandoraea novymonadis" endosymbiont. mBio. 2021; 12(4): e0160621. doi: 10.1128/mBio.01606-21.
