# Supplementary material for: Chromosome level genome assembly of the World Health standards Leishmania (Viannia) guyanensis M4147 and L. (V.) shawi M8408 using a hybrid sequencing approach
Source: Mem Inst Oswaldo Cruz. 2026 Jun 19;121:e250270. doi: 10.1590/0074-02760250270 (PMC13281984; doi:10.1590/0074-02760250270)
Supplement: Peer review history

# Reviewer report: FIRST REVIEW ROUND - REVIEWERS' COMMENTS

## REVIEWER #1

I find the work of Tullume Vergara at al. an important addition to the growing list of well-assembled Leishmania genomes and I think it will benefit the community. Nevertheless, I do have a few suggestions on how to improve presentation to make it clearer (see below). In principal, it may be interesting to see 2 additional analyses, but they may go beyond the scope of this short communication: a) analysis of telomeres and sub-telomeric sequences; b) analysis of hybridization.

Comments:

1) Reference 2 is strange. Suggest doi:10.1098/rsob.200407.

2) Reference 3 is simply wrong.

3) How the 3 intron-containing genes were annotated in the "intronless" mode?

4) I suggest not to abbreviate the first word in a sentence: Leishmania instead of L.

## REVIEWER #2

a) Adequacy of the abstract:

The abstract is concise and clearly states the objective, methods, and main outcome (chromosome-level assemblies of *L. (V.) guyanensis* and *L. (V.) shawi*).

b) Originality and importance of the contribution for the development of the field of study:

The study contributes valuable reference genomes for two WHO standard Leishmania strains, which are important for comparative and evolutionary studies in the Viannia subgenus. While similar genomes have been published previously, the chromosome-level assemblies using a hybrid approach represent an improvement in completeness and contiguity. The originality lies mainly in the technical achievement rather than in new biological discoveries. Expanding the discussion to include biological implications would enhance the manuscript's significance.

c) Methodology, results and discussion:

The methodology is appropriate, up-to-date, and well described, using current tools (Flye2, RagTag, Pilon, BUSCO, SyRI, GenomeScope). The assembly metrics are solid and the completeness values indicate high-quality genomes. The Results and Discussion are predominantly descriptive. The authors could better explore comparative genomic aspects, gene family differences, or functional annotations. The synteny and CNV analyses are mentioned but not fully interpreted in a biological context. Including a brief discussion on how structural variations or chromosome 31 aneuploidy relate to Leishmania adaptation or pathogenicity would make the work more impactful.

d) References:

The references are comprehensive and include all essential citations for tools and comparative studies.

e) Figures and tables:

The figures are of good quality and adequately illustrate the main results. The tables are clear and informative.

## AUTHORS' RESPONSE TO THE REVIEWERS

**Manuscript Number:** MIOC-2025-0270

Dear Editor-in-Chief

We sincerely thank the editor and reviewers for their thorough and insightful review of our manuscript. We have carefully considered all comments and have made the necessary revisions to address the concerns raised. Below, we provide detailed responses to each comment.

Sincerely,

João Marcelo Pereira Alves

## Reviewer 1

**1) Reference 2 is strange. Suggest doi:10.1098/rsob.200407.**

Thank you for this valuable suggestion. We have updated the citation to a more appropriate and recent reference. The reference in the introduction, line 40, has been replaced accordingly (reference 2. Kostygov et al., 2021).

**2) Reference 3 is simply wrong.**

We thank the reviewer for pointing this out. The reference has been corrected.

**3) How the 3 intron-containing genes were annotated in the "intronless" mode?**

We thank the reviewer for this important question. The three intron-containing genes were manually annotated based on similarity searches and profile-based methods, bypassing the "intronless" option for these specific cases. This has been clarified in the Methods section.

**4) I suggest not to abbreviate the first word in a sentence: Leishmania instead of L.**

We thank the reviewer for this suggestion and have revised the manuscript accordingly throughout the text.

## Reviewer 2

**a) Adequacy of the abstract:**

We sincerely thank the reviewer for this positive and encouraging feedback on the manuscript.

**b) Originality and importance of the contribution**

Producing chromosome-level assemblies for *L. (V.) guyanensis* and *L. (V.) shawi* is valuable, especially using WHO reference strains. These genomes fill a gap in Viannia species and will support future comparative genomics, epidemiology, and drug-target exploration.

However, the manuscript focuses almost exclusively on generating and describing the assemblies. There is limited biological insight, in-depth comparative analysis, or interpretation of the structural variation detected.

Answer:

We sincerely thank the reviewer for this positive and encouraging feedback on the all manuscript.

**c) Adequacy of methodology, results, and discussion**

Methodology:

The sequencing and assembly pipeline is appropriate and uses standard, well-established tools (Flye, RagTag, Pilon, BUSCO, SyRI). Methods are described in sufficient detail, though some parameters (e.g., polishing iterations) could be clarified. Quality control and read statistics are presented clearly.

Answer:

We thank the reviewer for their valuable input. In response, we have made the following enhancements to strengthen the manuscript: Methodology: We employed a standardized pipeline with the most current assembly and annotation tools to ensure high-quality genome references, presented as a short communication for rapid community access. Their suggestion was added to line 92.

Results:

The results section mostly presents assembly statistics, BUSCO scores, read depth somy, and structural variants. These are useful, but the manuscript would benefit from:

A clearer rationale for choosing *L. braziliensis* as the scaffolding reference.

I suggest complementing Figure 2 with a circus plot.

A discussion of large-scale structural variation across species and its biological meaning.

More detailed analysis of multigene family content (briefly mentioned but not developed).

A stronger examination of chromosome 31 aneuploidy, a known feature in Viannia.

Answer:

We selected *L. braziliensis* strain M24904 as the reference genome because it is a well-characterized strain with a high-quality chromosome-level assembly. This strain has been widely used in comparative genomic studies, providing a reliable framework for analysis. Additionally, *L. braziliensis* is one of the closest phylogenetic relatives of *L. shawi* and *L. guyanensis* within the Viannia subgenus, making it an appropriate and biologically relevant reference for our study.

We have expanded the Discussion section to provide a more detailed interpretation of the key findings of this study. While some suggestions could not be incorporated due to time constraints and the specific scope of our manuscript, we have carefully considered the majority of the reviewer's comments and addressed them accordingly. In particular, we have revised and clarified relevant sections to resolve the concerns raised and improve the overall clarity of the manuscript.

Discussion:

The discussion is brief and fails to contextualize the findings within current knowledge of Leishmania genomics. As a research article, it requires deeper interpretation. As a genome-resource paper, it is acceptable, but it could still be strengthened.

Answer:

Also, we added a discussion section in our manuscript intent to fill some gaps in the first version: For example: Aneuploidy; We added a new subsection discussing aneuploidy in Leishmania, addressing chromosomal copy number variations and their biological implications. Phylogenomics; We incorporated a phylogenomics-based analysis to elucidate evolutionary relationships within the Viannia subgenus, filling a key knowledge gap (Materials and methods, and results).

**d) References**

References are generally adequate and include essential genomic and methodological citations.

A few points:

Reference (2) is to Wikipedia and should be replaced with an authoritative taxonomic source.

The manuscript would benefit from citing more recent papers on the Leishmania (Viannia) genome and comparative genomics studies.

Answer:

Thank you for this valuable suggestion. We have updated the citation to a more appropriate and recent reference. The reference in the introduction, line 40, has been replaced accordingly (reference 2. Kostygov et al., 2021).

Comments from the Editor:

For JBIEOV000000000.1 Leishmania guyanensis, for example, there appear to be 47 contigs (JBIEOV010000001–JBIEOV010000047), plus an additional ~30 kb sequence (CM124108) identified as kDNA. This structure does not align with the descriptions provided in the text or in the tables, and clarification is required.

It is also important to include transcriptomic evidence to support the gene annotation.

Answer:

We appreciate your careful review and for identifying the error in the count reported in our manuscript. We have corrected this issue accordingly and thank you for noting the discrepancy in the NCBI submission.

We did not perform transcriptomic sequencing, as it was beyond the scope and primary objectives of this study. However, we agree that incorporating transcriptomic data would significantly improve gene annotation quality and enhance the accuracy of gene family characterization. This is an important consideration for future work.

Although categorized as a Research Article, the manuscript reads more like a genome announcement or dataset description, with limited hypothesis-driven content or biological analysis. We encourage the authors to expand the discussion to better highlight biological relevance.

General answer:

Dear Editor, we sincerely thank you for your careful and constructive evaluation of our manuscript. Our primary aim was to provide a concise genome sequence report (Short Communication) for these medically important species, for which genomic resources remain limited—particularly in Latin America and Brazil. We believe that making these data available will help address an important gap and support future research in the region. Here is an example of "short-comunication" in MIOC journal: https://www.scielo.br/j/mioc/a/F6fxwygbnp3sXGHcBztcqLd/?format=html&lang=en. Also, we added a "discussion section" in our manuscript, with the intent to fill some gaps found in the first version.

# Reviewer report: REVIEWERS' COMMENTS

## REVIEWER #1

No comments.

## REVIEWER #2

No comments.

## REVIEWER #3

No comments.

## Comments from the Editor:

The manuscript can be approved in its current version.
